# Natural Language Processing in Nursing Practice for Clinical Reasoning and Workflow: Scoping Review

**DOI:** 10.2196/94778

**Published:** 2026-10-16

**Authors:** Keisuke Iwase, Taiki Teshima, Miyae Yamakawa, Yasushi Takeya

**Affiliations:** 1 Faculty of Nursing, School of Health Sciences, Fujita Health University, Toyoake, Aichi, Japan; 2 Department of Evidence-Based Clinical Nursing, Division of Health Sciences, Graduate School of Medicine, Osaka University, 1-7 Yamadaoka, Suita, Osaka, Japan, 1 6-6879-2543; 3 Faculty of Nursing, Kansai Medical University, Hirakata, Osaka, Japan

**Keywords:** clinical decision support, nursing documentation, natural language processing, scoping review, AI

## Abstract

**Background:**

Recent advancements in digital health technologies have led to the accumulation of large volumes of unstructured text data, particularly in electronic health records. Given that nursing documentation frequently includes detailed descriptions of daily living activities and psychosocial contexts, existing medical natural language processing (NLP) tools may not be directly applicable without adaptation. Moreover, the integration of standardized nursing terminologies into NLP systems remains limited. Although NLP has the potential to support risk detection, information management, and care planning, its practical applications in nursing have not yet been systematically mapped.

**Objective:**

This scoping review aimed to map the practical applications of NLP in nursing practice, with a specific focus on real-world clinical use, particularly for assessment, documentation, and interventions, and to identify the study characteristics, application domains, outcomes, and challenges.

**Methods:**

Following the Joanna Briggs Institute methodology and PRISMA-ScR (Preferred Reporting Items for Systematic Reviews and Meta-Analyses Extension for Scoping Reviews) guidelines, a systematic search was conducted across PubMed, CINAHL, the Cochrane Library, Scopus, Embase, and Ichushi-Web in October 2023 and June 2025. The population-concept-context framework (population: nurses; concept: NLP applications; context: clinical settings) was used to guide study selection. Two independent reviewers screened 2889 unique records and reviewed the full texts of 72 studies. Data were descriptively synthesized and thematically classified.

**Results:**

Six studies met the inclusion criteria, indicating that empirically evaluated applications of NLP in real-world nursing practice remain limited. NLP applications were categorized into three domains: (1) text summarization and generation, (2) information extraction and classification, and (3) real-time detection and screening. NLP was applied across multiple stages of nursing practice, including handovers, assessment, care planning, continuity of care, and patient safety management. Notably, NLP was used as a form of augmented intelligence to complement, rather than replace, nurses’ clinical judgment and to support clinical decision-making. These findings also highlight a substantial gap between rapid technological advances and the limited implementation of NLP in real-world nursing practice.

**Conclusions:**

This review highlights the substantial gap between rapid technological advances and the limited implementation of NLP in real-world nursing practice. NLP may help reduce manual review workload, support early risk identification, and facilitate individualized care planning in selected nursing contexts. However, direct empirical evidence regarding its effects on nursing workflows, nurses’ clinical decision-making, and patient-level outcomes remains limited. Future research should prioritize real-world implementation studies and evaluate nurses’ acceptance, adoption barriers, workflow integration, and the influence of NLP on clinical decision-making.

## Introduction

Recent advances in information technology have resulted in the daily generation of vast amounts of health care–related data through digital systems, such as electronic health records (EHRs). EHRs comprise longitudinal data that are routinely collected in clinical settings, including patient demographic information, vital signs, clinical documentation, insurance claims data, patient-reported outcomes, and data obtained from home-based monitoring devices [1]. A previous review examined applications of the Internet of Things in health care and demonstrated that the integration of Internet of Things devices with cloud computing and AI technologies enables real-time remote monitoring and data-driven clinical insights, thereby contributing to transformative changes in health care delivery [2]. These technological developments have made the acquisition of clinical data easier. Consequently, the effective use of health care data in medical and nursing practice has become increasingly important, highlighting the need for advanced analytical approaches such as natural language processing (NLP).

NLP enables computers to analyze text and speech in natural language and transform unstructured information into structured data that can be computationally processed [3]. In health care, NLP has attracted increasing attention as a means of extracting meaningful information from text data in EHRs, and research in this field has expanded rapidly in recent years [4,5]. However, a substantial proportion of health care data, including EHRs, consists of unstructured text, which is estimated to account for approximately 80% of all medical data [6].

Although these textual data contain rich information about patients’ conditions, clinical trajectories, and interventions, they are more difficult to analyze and use than structured data [7]. Specifically, nursing documentation generally describes patients’ activities of daily living and psychosocial contexts in greater detail than medical diagnoses, which limits the direct applicability of existing medical NLP tools to nursing records. Existing NLP tools are primarily optimized for diagnostic terminologies and may not adequately support the analysis of nuanced expressions, domain-specific vocabulary, and clinical reasoning inherent in nursing documentation. Furthermore, variations in documentation styles across institutions and individual nurses pose a fundamental challenge to the standardization of NLP systems. The integration of standardized nursing languages—such as NANDA-International (NANDA-I), the Nursing Outcomes Classification, and the Nursing Interventions Classification—into EHRs enables the systematic documentation of nursing practice and facilitates the visualization and evaluation of nursing expertise and professional contributions [8].

Previous studies [9] have reported various applications of NLP in health care, including patient risk prediction and the analysis of clinical conversations. In nursing, NLP has also been applied to tasks such as symptom detection from home care records [10], hospitalization risk prediction based on nursing documentation [11], extraction of symptom-related information from EHRs [12], and fall risk prediction [13]. However, previous reviews have reported that most studies have focused primarily on technical aspects, such as disease classification and algorithm development, with limited attention to practical implementation for clinical interpretation and nursing assessment [12,14].

In recent years, the application of generative AI and large language models (LLMs) in health care has expanded rapidly, attracting considerable attention. However, a gap between technological advancement and real-world implementation has also been highlighted. According to a recent review [15], numerous LLM-related studies in health care have been published between 2023 and mid-2024. Nevertheless, most of these studies have focused on tasks such as question answering, with only limited research addressing clinical workflow support. Furthermore, concerns have been raised regarding the methodological rigor of many studies focusing on these emerging technologies [16]. Therefore, despite rapid advancements in AI, structural barriers to integration into clinical practice remain unresolved. Accordingly, rather than focusing on emerging technologies such as LLMs or ambient AI, this study adopts an implementation-oriented scoping review that prioritizes NLP applications implemented and evaluated within real-world nursing workflows.

Therefore, this scoping review aimed to comprehensively map how NLP has been applied in nursing practice, particularly to support nursing assessment, documentation, and interventions, with a focus on real-world clinical implementation. The present study also aimed to characterize previous research, identify application domains, and clarify reported outcomes and challenges. Specifically, we sought to (1) categorize the distinct types and roles of NLP approaches, (2) identify their impact across various stages of the nursing process, and (3) summarize the reported outcomes and barriers to implementation. By doing so, this study provides foundational insights for integrating “augmented intelligence” into professional nursing care.

## Methods

### Study Design

This scoping review followed the Joanna Briggs Institute methodology [17] and the PRISMA-ScR (Preferred Reporting Items for Systematic Reviews and Meta-Analyses Extension for Scoping Reviews) guidelines [18] (Checklist 1). This design was selected to map the existing evidence base and identify gaps in the evolving domain of NLP in nursing practice.

### Search Strategy

The literature search was conducted using PubMed, CINAHL, the Cochrane Library, Scopus, Embase, and Ichushi-Web (Japan). The initial search was conducted on October 5, 2023. In the updated search, performed on June 13, 2025, the phrase “data mining” was added to enhance the search’s sensitivity and comprehensiveness. The comprehensive search strategies for each database are tabulated in Multimedia Appendix 1. Equivalent search strategies were adapted for each database, as appropriate. The search strategy was designed to prioritize foundational NLP and data mining approaches with established evidence of clinical integration, reflecting the implementation-oriented scope of this review. Accordingly, the review focuses on empirically evaluated applications within nursing workflows, rather than capturing the broad spectrum of emerging technologies such as generative AI or LLMs.

### Eligibility Criteria

Using the Joanna Briggs Institute population–concept-context framework, the inclusion criteria were as follows: nurses (population), NLP applications in nursing practice (concept), and clinical settings (context). Eligible studies were peer-reviewed original articles in English or Japanese that described NLP applications in nursing practice (eg, assessment, documentation, or interventions). The eligibility criteria were specifically defined to identify studies describing the use of NLP within real-world nursing practice and clinical workflows. Studies were excluded if they (1) focused solely on technical model performance or diagnostic accuracy without clinical application or nursing involvement, (2) focused on physician-centered or other nonnursing applications without explicit involvement of nurses, or (3) were gray literature (eg, conference abstracts or proceedings) lacking sufficient methodological detail. Among studies addressing similar topics (eg, automated identification from clinical or radiology reports), only those that described applications within nursing-related clinical workflows (eg, assessment, documentation, or interventions) were included.

### Study Selection

Two independent reviewers screened studies in 2 stages: screening of titles and abstracts, followed by full-text review. Disagreements were resolved through consultation with a third author. 19-21,19-24

### Data Extraction and Analysis

Data were extracted using a standardized form (see Multimedia Appendix 2) that included author, year, country, design, population, nursing practice domain, NLP techniques, performance metrics, human intervention points, and outcomes. The extracted data were descriptively synthesized. NLP techniques were categorized according to their methodological approach (eg, rule-based and machine learning), and applications were thematically classified according to clinical context (assessment, documentation, and interventions). Common themes among outcomes and challenges were aggregated.

### Ethical Considerations

This study is a scoping review based on previously published literature and did not involve human participants. Therefore, ethics approval was not required in accordance with the authors’ institutional policies.

## Results

### Study Selection Results

From the 1535 unique records in the initial search, 24 studies underwent full-text review, and 3 studies were included [19-21]. The updated search yielded an additional 1354 unique records and 48 full-text reviews, adding 3 more studies. Overall, 2889 unique records were screened, 72 full-text studies were reviewed, and 6 studies were included [19-24]. Hand searching the 11 references from relevant reviews identified no further studies.

As illustrated in the PRISMA (Preferred Reporting Items for Systematic Reviews and Meta-Analyses) flow diagram, most of the records excluded during the title and abstract screening and the full-text review stages focused on the technical development or performance evaluation of NLP models and did not describe nursing practice or clinical workflow applications. The screening and selection process was conducted systematically and transparently based on predefined inclusion and exclusion criteria (Figure 1).

**Figure 1.**
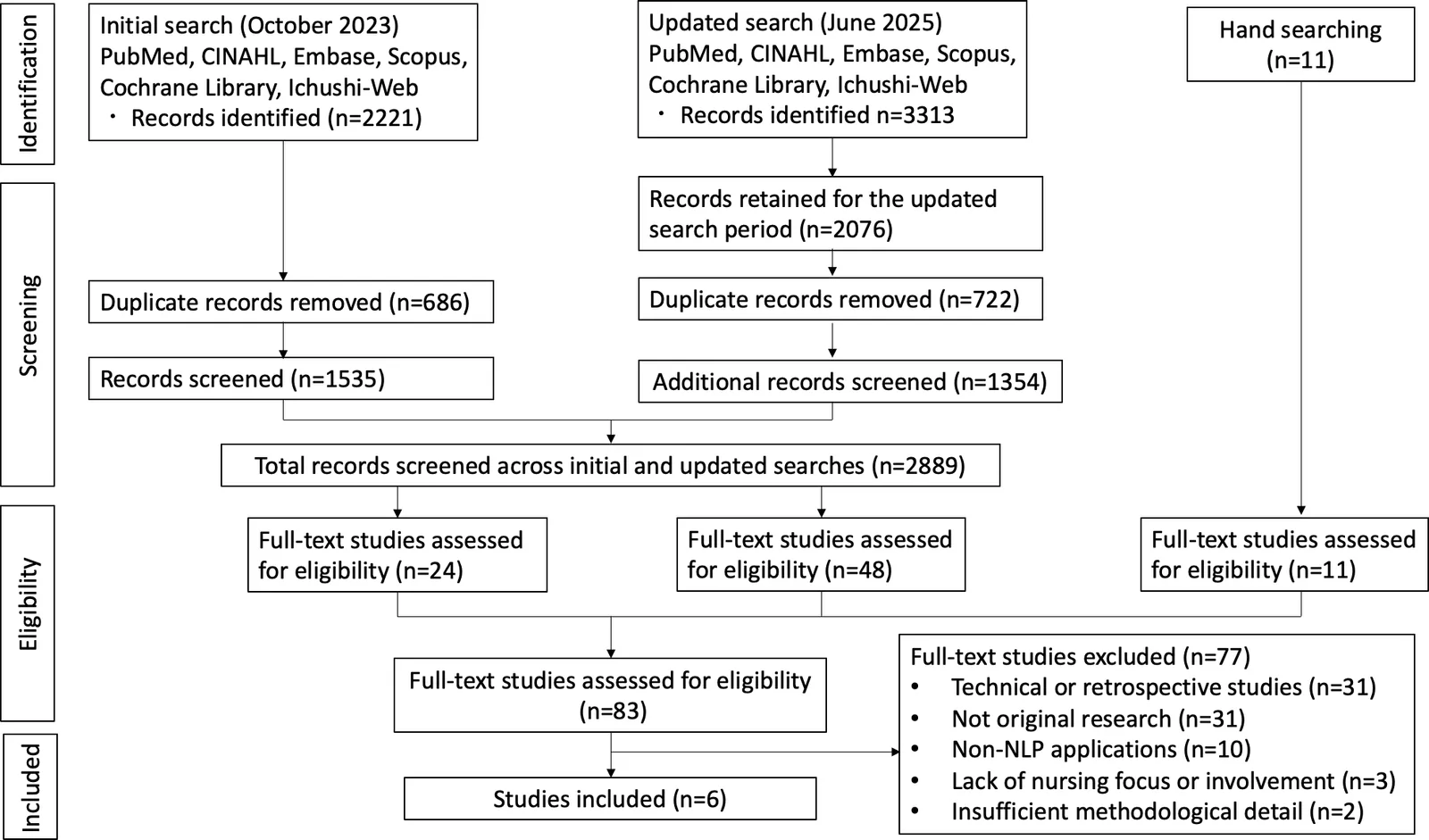
PRISMA (Preferred Reporting Items for Systematic Reviews and Meta-Analyses) flow diagram of study selection.

### Characteristics of Included Studies

Table 1 summarizes the characteristics of the included studies. The examined studies were published between 2012 and 2025 [19-24]. Four studies were conducted in the United States [19,21,23,24], 1 in the United Kingdom [22], and 1 in Taiwan [20]. The study designs included a pilot implementation study [20], retrospective and prospective implementation evaluation studies [19,21-23], and an observational implementation study [24].

**Table 1.** Characteristics of included studies.

Author (year)	Setting	Population	Study design	Data sources
Hunter et al [22], 2012	NICU^a^	Neonates	Prospective	EHR^b^ (physiological data, notes)
Evans et al [19], 2016	Emergency department	Heart failure patients	Pre-post	EHR (physiological data, reports)
Tang et al [20], 2019	LTCF^c^	Older adults	Pilot	Nursing records
Boitano et al [21], 2023	Academic hospital	AAA^d^	Retrospective and prospective	Radiology reports (EHR)
Waltzman et al [23], 2024	Community	HAV^e^ cases	Cross-sectional	Nursing notes
Nguyen et al [24], 2025	Integrated system	Patient safety cases	Observational	EHR notes

a NICU: neonatal intensive care unit.

b EHR: electronic health record.

c LTCF: long-term care facility.

d AAA: abdominal aortic aneurysm.

e HAV: health care–associated violence.

The primary data sources used across these studies consisted of unstructured text documented in EHRs, including clinical notes, nursing notes, incident reports, and handover records. The NLP techniques involved rule-based approaches [22-24], machine learning–based classification and prediction methods [19,20,23], and natural language generation [22]. Notably, all included studies implemented NLP as part of existing clinical systems involving nurses, rather than as a stand-alone technical intervention. For a more comprehensive description of study characteristics, including detailed setting information and specific data extraction methods, see Multimedia Appendix 3.

### Categorization and Roles of Applied NLP Approaches

#### Overview

The applied NLP approaches were further categorized into 3 distinct types based on their clinical roles (Table 2). For a more comprehensive description of these approaches, see Multimedia Appendix 4.

**Table 2.** Application of natural language processing (NLP) in nursing practice.

Author (year)	NLP task (type)	Role of NLP	Direct impact on nursing	Key challenges
Hunter et al [22], 2012	Summarization	Automated summarization of clinical information	Handover and information sharing	Domain-specific (respiratory and cardiovascular)
Tang et al [20], 2019	Summarization	Care plan generation based on clinical data	Support for chronic disease management	Data privacy and security
Evans et al [19], 2016	Extraction	Identification of high-risk patients from EHR^a^ data	Clinical decision support	Multicenter validation needed
Boitano et al [21], 2023	Extraction	Identification of patients with AAA^b^ from radiology reports	Population health and nurse navigation	Dependence on explicit documentation
Waltzman et al [23], 2024	Extraction	Extraction of violent incidents from nursing notes	HAV^c^ prevention and safety management	Manual review burden
Nguyen et al [24], 2025	Detection	Real-time triage of patient safety concerns	Telephone nursing triage	Limited infrastructure

a EHR: electronic health record.

b AAA: abdominal aortic aneurysm.

c HAV: healthcare-associated violence.

#### Type 1: Text Summarization and Generation

This approach involves generating standardized information from free-text nursing documentation to serve specific clinical purposes. One system [22] used natural language generation techniques to automatically produce standardized nursing shift summaries from EHR notes. This system reduced information loss during handovers and improved the quality of clinical information transfer. Similarly, another system [20] was developed to integrate historical nursing records and admission assessment documents to generate individualized nursing care plans. Notably, the system automatically retrieved up-to-date evidence, such as best practice guidelines, from web-based sources and incorporated this information into the generated plans, supporting the integration of evidence-based practice into routine nursing care.

#### Type 2: Information Extraction and Classification

This approach focuses on systematically extracting and identifying information related to specific diseases, risks, or events from unstructured nursing documentation or clinical reports. One study [19] applied a proprietary NLP algorithm to analyze comprehensive medical records, significantly improving the sensitivity for identifying patients with heart failure. Implementation of the system was associated with reductions in clinicians’ diagnostic time and the 30-day mortality rate. Similarly, an existing NLP system [21] was used to automatically identify patients with abdominal aortic aneurysms (AAA) from radiology reports, enabling reliable referral of patients requiring follow-up and resulting in timely surgical interventions. Furthermore, an NLP-based method [23] extracted health care–associated violence (HAV) events from nursing notes. This facilitated high-accuracy identification of violent incidents that were frequently underreported in conventional incident reporting systems, thereby enhancing the visibility of workplace safety issues.

#### Type 3: Real-Time Detection and Screening

This approach involves the real-time analysis of free-text data to prioritize triage decisions or identify patient safety concerns. A real-time analysis system [24] examined appointment notes and triage documentation during outpatient scheduling to identify safety concerns. When detecting high-risk signals, nurses conducted immediate telephone follow-ups. This mechanism served as a clinical “safety catch” by supporting the early identification and follow-up of serious safety concerns. Furthermore, the system reduced the number of cases requiring manual review by approximately 80% (from a monthly average of 4215 to 705 cases), demonstrating improved operational efficiency.

### Applications and Outcomes Across the Stages of the Nursing Process

The applications of NLP identified in the included studies were categorized according to the nursing process stages (Tables 1 and 2).

#### Information Sharing and Handover

For information sharing during shift changes, NLP-based automated summarization supported nurses by presenting patient information in a structured and comprehensible format. One study [22] reported that automatically generated nursing handover summaries were highly rated by clinicians, with 90% considered easy to understand, 70% considered accurate, and 59% considered helpful. These findings suggest that NLP-mediated summarization may support effective information sharing during handovers.

#### Assessment and Discharge Planning

The use of NLP enabled early identification of high-risk patients and contributed to the acceleration of discharge support processes. An integrated NLP system [19] was used to analyze unstructured clinical notes alongside existing structured data (such as *International Classification of Diseases* codes), improving the sensitivity of patient identification for heart failure. This approach reduced clinicians’ chart review time (10 vs 40 min), facilitating earlier assessment and intervention.

#### Care Planning

NLP supported the more rapid development of individualized care plans. One study [20] reported a reduction in the time required for care planning from 7 to 4 days (a 42.8% improvement) and an increase in the acceptability of these plans from 39% to 78%. The system also supported more consistent care planning, particularly among less experienced staff.

#### Follow-Up and Continuity of Care

Continuous system-based monitoring optimized nurse navigation and enabled proactive outreach. A large-scale analysis [21] identified 6495 patients with AAA from 6.3 million radiology reports and facilitated 29 follow-up outpatient visits for patients whose surveillance had lapsed, as well as 4 surgical interventions for 3 patients. These outcomes helped improve adherence to guideline-recommended surveillance.

#### Safety Management and Surveillance

NLP enhanced the visibility of safety threats, which were often overlooked by conventional reporting systems. Using an NLP-assisted approach [23], HAV events were extracted from nursing notes with a sensitivity of 96.8%. In addition, a real-time safety screening [24] facilitated the identification of serious concerns and reduced the number of cases requiring manual review by approximately 80% (from a monthly average of 4215 to 705 cases), demonstrating improved operational efficiency.

#### Outcomes and Challenges of NLP Implementation

Across the included studies, the primary reported benefits of NLP implementation included reduced nursing workload, enhanced identification of specific risks or target populations, and support for nurses’ clinical decision-making (Table 2). Specifically, the studies reported standardized handovers and reduced information loss [22], earlier identification of high-risk patients and acceleration of discharge planning [19], improved efficiency of care plan development and integration of up-to-date clinical knowledge [20], proactive follow-up of patients with AAA who have not received recommended surveillance [21], increased visibility of HAV events [23], and facilitation of concern identification with a substantial reduction in nurses’ time spent on manual record review [24]. Notably, these functions did not replace nurses’ decision-making; rather, NLP operated as a form of augmented intelligence that complemented professional judgment and supported the translation of insights into appropriate nursing interventions.

All included studies reported notable challenges and limitations. Some studies were conducted within a single institution [19], emphasizing the need for external validation across diverse clinical settings. Studies using rule-based or keyword-dependent approaches [21,22,24] reported that inaccuracies in documentation and variability in language use resulted in false positives or missed cases. To mitigate these technical limitations and the risk of erroneous outputs, the included studies implemented human-in-the-loop mechanisms to safeguard clinical judgment. These mechanisms functioned as important safeguards against algorithmic rigidity and potential errors in automated classification, ensuring that qualified clinicians, such as specialist nurses or nurse navigators, retained responsibility for clinical decisions by reviewing and validating NLP outputs (Table 2). Additionally, several studies emphasized that translating NLP-derived information into actual clinical interventions necessitated the availability of dedicated nursing personnel, such as nurse navigators or specialized teams [21,23]. Overall, these findings suggest that NLP complements rather than replaces nursing judgment. By structuring narrative data, NLP may facilitate informed decision-making. Therefore, future NLP systems should be designed as robust human-in-the-loop tools that empower nurses as final arbiters of clinical safety, ensuring seamless integration into nursing workflows.

## Discussion

### Summary of Findings

In this scoping review, only 6 studies [19-24] met the inclusion criteria, highlighting a substantial implementation gap between the rapid advancement of NLP technologies and their integration into real-world nursing practice. The limited number of eligible studies is an important finding in itself. Rather than focusing solely on whether NLP can technically process nursing documentation, this review highlights the broader question of why implementation-oriented NLP research in nursing remains so limited. The findings of this review suggest that this implementation gap reflects interrelated technical, organizational, and professional and cultural barriers to implementation.

### Factors Contributing to the Implementation Gap

#### Technical Barriers

Nursing documentation often consists of heterogeneous free-text narratives describing patients’ health status, symptoms, functional status, and other contextual information, which makes standardization challenging [8,14]. Standardized nursing terminologies are therefore fundamental to ensuring the comparability, interoperability, and secondary use of nursing data [8]. However, a recent review found that the use of standardized nursing terminologies in nursing NLP research remains limited [14]. Furthermore, a recent study reported that nurses perceived institutional and technical barriers to implementing NANDA-I, Nursing Interventions Classification, and Nursing Outcomes Classification in clinical practice [25]. These limitations in the adoption of standardized nursing terminologies may hinder the development and external validation of NLP systems for nursing documentation, thereby limiting their implementation and generalizability. In addition, the limited representation of standardized nursing terminologies in NLP training datasets may constrain the ability of NLP models to recognize nursing-specific concepts, relationships, and clinical reasoning patterns.

#### Organizational Barriers

EHR systems continue to face challenges related to data access, interoperability, privacy protection, and data governance [1,26]. Access to nursing record datasets may also be restricted by institutional ethics review requirements, data-use approval processes, and concerns regarding the secondary use of sensitive clinical documentation. Similar implementation barriers, including inadequate technical support, poor system integration, and usability issues, have also been identified in a scoping review of EHR implementation [27]. Furthermore, insufficient involvement of end users during the planning, development, and implementation of EHR systems has been recognized as a common implementation barrier [27]. These organizational challenges may hinder the integration of NLP into routine nursing workflows and limit its implementation in clinical practice.

#### Professional and Cultural Barriers

Although physician-centered structured data have been the primary focus of EHR and NLP research, nursing documentation may have been underused. This imbalance may partly explain why implementation studies of NLP in nursing remain limited. Consistent with this concern, a previous review [28] found that nurses’ involvement was limited, particularly during the early phases of AI development, and that ethical considerations related to the implementation and use of AI in nursing received limited attention. In addition, publication bias may have contributed to the underreporting of unsuccessful implementation efforts or negative findings, thereby skewing the available evidence toward successful cases. Taken together, these professional and cultural barriers may help explain why implementation-oriented NLP research in nursing remains limited. Addressing these barriers may require involving nurses as coinvestigators throughout the development and evaluation of NLP systems to ensure that these technologies are aligned with nursing practice.

### Implications for Implementation

Unlike previous reviews, which have predominantly focused on the technical aspects of NLP or broader implementation and ethical issues related to AI in health care [12,14,29], this review examined how NLP has been integrated into nursing workflows and the extent to which nurses are involved in these systems in real-world clinical settings. Although recent interest has shifted toward LLM-based applications, empirical evidence regarding the implementation of these emerging technologies remains limited. Consistent with previous comprehensive reviews [28], there is limited empirical evidence regarding the effects of NLP on clinical outcomes, nurses’ decision-making, and integration into routine nursing practice. This pattern is also evident in more recent research. For example, a recent review [15] reported that only 12.5% of 415 studies using advanced LLMs focused on clinical workflow support, whereas most applications addressed clinical decision support or patient information support. Similarly, although ambient AI technologies have recently received substantial attention, peer-reviewed empirical evidence regarding their implementation in nursing practice remains limited. Notably, although some studies have reported usability or perceived helpfulness [22], nurse acceptance and implementation barriers have rarely been examined explicitly, highlighting another important gap in the current evidence base. These findings suggest that the successful implementation of NLP in nursing depends not only on technological performance but also on organizational support, professional engagement, and well-defined implementation processes.

### NLP as Augmented Intelligence

Previous reviews have noted that health care NLP has primarily emphasized algorithm development, data extraction, and classification tasks, with relatively limited attention to supporting nurses’ judgment, decision-making, and care processes [12,14]. Specifically, one review [12] reported that many studies primarily used NLP for classification, whereas an integrative review [14] highlighted the need for improved evaluation, standardization, and broader application of NLP in nursing. Building on these observations, this review shifted the focus from technical accuracy alone to empirical evidence of how NLP is used in nursing practice and how it may support clinical decision-making. The included studies suggest that NLP may reduce manual review workload, improve patient identification, and facilitate clinical decision-making in selected nursing contexts [19,21,24]. Collectively, these findings indicate that NLP may support clinical workflows by functioning as an analytical tool and a decision support resource. However, direct empirical evidence regarding patient outcomes remains limited.

These findings suggest that NLP should not be conceptualized as a technology that fully automates nurses’ clinical judgment. Rather, it may be better understood as a form of augmented intelligence that complements and supports cognitive processes in nursing practice. In particular, NLP may support clinical reasoning by processing large volumes of unstructured data during the early stages of assessment and information processing [30]. This perspective aligns with the principle that clinical decision support systems should be integrated into clinical workflows while preserving professional judgment [31]. The reported usefulness of automatically generated summaries [22] is consistent with this perspective and with the broader concept of the convergence of human and artificial intelligence [32].

Previous studies have identified several challenges associated with clinical decision support systems, including omission and commission errors [33] and concerns regarding data security and patient privacy [20]. As NLP becomes increasingly incorporated into clinical decision support and nursing workflows, related risks, including automation bias, warrant careful consideration. However, empirical evidence on how these challenges affect the implementation of NLP in nursing practice remains limited.

Accordingly, future implementation efforts should focus on the safe and responsible integration of NLP into nursing practice. Because NLP applications process sensitive personal health information, robust privacy protection and data security are essential [20,34,35]. Previous studies have also emphasized the importance of explainability and responsible AI implementation [36]. Building on these considerations, future research should explore strategies to improve AI literacy among nurses and thereby facilitate the responsible implementation of NLP in clinical practice.

Taken together, these findings suggest that NLP should be considered not merely as a technical tool but as part of clinical systems that involve nurse engagement. This perspective is conceptually aligned with the Non-Adoption, Abandonment, Scale-Up, Spread, and Sustainability framework [26], particularly regarding NLP adoption, integration into routine clinical workflows, and long-term sustainability in nursing practice. The technical, organizational, and professional barriers identified in this review may represent key challenges to the adoption, routine integration, and long-term sustainability of NLP in nursing practice. This review, therefore, provides an implementation-oriented perspective that may guide future research and support the integration of NLP into nursing practice.

### Limitations

This scoping review has several limitations. First, the literature search was restricted to studies published in English and Japanese, potentially introducing language bias. Second, the number of databases searched was limited, and gray literature, including doctoral dissertations, conference proceedings, and unpublished reports, was excluded. Consequently, some relevant studies may have been missed. Furthermore, as this review specifically focused on NLP applications in nursing practice, it excluded studies that did not explicitly involve nurses in clinical workflows (eg, physician-centered applications). Future reviews should incorporate broader database coverage and gray literature to provide a more exhaustive overview of the field. Third, the number of included studies was small, reflecting the limited implementation of NLP in nursing practice and the early stage of implementation-oriented research in this field. Accordingly, this review provides a map of current research trends rather than definitive conclusions, and further empirical studies are warranted as the field evolves. Fourth, the search strategy was predominantly designed to capture foundational NLP and data mining techniques. Therefore, it may not have fully captured the most recent studies focusing on generative AI and ambient AI technologies, which have proliferated rapidly in recent years. Given the recent surge in studies using these technologies for nursing documentation, summarization, and care planning, this review’s findings may not fully reflect this emerging body of work. Future studies should compare traditional NLP approaches with newer generative AI technologies to explore their distinct impacts on nursing practice. Fifth, a geographic bias exists, as the included studies were conducted in a limited number of regions, specifically the United Kingdom, the United States, and Taiwan. Consequently, the findings may have limited generalizability to diverse nursing environments with different health care systems and cultural contexts. Finally, in accordance with the methodological nature of a scoping review, this study did not include a formal appraisal of the methodological quality or the risk of bias of the included studies. Therefore, caution should be exercised when interpreting the findings. Future research should incorporate systematic quality assessment to strengthen the evidence base and validate the conclusions drawn.

### Conclusions

This scoping review suggests that NLP may support nursing practice by extracting and generating clinically relevant information from unstructured nursing-related text. However, empirical evidence regarding its implementation in real-world nursing practice remains limited. Rather than replacing professional judgment, NLP may function as a form of augmented intelligence that supports nurses’ clinical decision-making. In selected nursing contexts, NLP may help reduce manual review workload, facilitate early risk identification, and support information processing. Nevertheless, direct empirical evidence regarding its effects on nursing workflows, nurses’ clinical decision-making, and patient-level outcomes is limited. Future research should examine nurses’ acceptance, adoption barriers, and implementation processes in real-world clinical settings.

Although previous studies have often focused on system development and technical performance, future research should prioritize implementation-oriented evaluations and assess how NLP influences nurses’ clinical decision-making in practice. To support the responsible integration of AI technologies into nursing practice, nurses may require sufficient AI literacy to critically appraise and interpret AI-generated outputs. Developing this knowledge and these skills may help nurses use AI technologies while maintaining professional judgment in an increasingly digital health care environment.

The principal contribution of this review is to identify the substantial gap between rapid technological advances and the limited implementation of NLP in real-world nursing practice, rather than to establish the effectiveness of NLP.

## Supplementary material

10.2196/94778 Multimedia Appendix 1 Search strategies for all databases.

10.2196/94778 Multimedia Appendix 2 Codebook.

10.2196/94778 Multimedia Appendix 3 Characteristics of included studies.

10.2196/94778 Multimedia Appendix 4 Application of natural language processing in nursing practice.

10.2196/94778 Checklist 1 PRISMA-ScR checklist.

## Abbreviations

AAA: abdominal aortic aneurysms
EHR: electronic health record
HAV: health care–associated violence
LLM: large language model
NLP: natural language processing
PRISMA-ScR: Preferred Reporting Items for Systematic Reviews and Meta-Analyses Extension for Scoping Reviews

## References

[1] Cowie MR, Blomster JI, Curtis LH (2017). Electronic health records to facilitate clinical research. Clin Res Cardiol.

[2] Li C, Wang J, Wang‎ S, Zhang Y (2024). A review of IoT applications in healthcare. Neurocomputing.

[3] Nadkarni PM, Ohno-Machado L, Chapman WW (2011). Natural language processing: an introduction. J Am Med Inform Assoc.

[4] Wu S, Roberts K, Datta S (2020). Deep learning in clinical natural language processing: a methodical review. J Am Med Inform Assoc.

[5] Wu H, Wang M, Wu J (2022). A survey on clinical natural language processing in the United Kingdom from 2007 to 2022. NPJ Digit Med.

[6] Murdoch TB, Detsky AS (2013). The inevitable application of big data to health care. JAMA.

[7] Durieux BN, Tarbi EC, Lindvall C (2022). Opportunities for computational tools in palliative care: supporting patient needs and lowering burden. Palliat Med.

[8] Jones D, Lunney M, Keenan G, Moorhead S (2010). Standardized nursing languages: essential for the nursing workforce. Annu Rev Nurs Res.

[9] Hossain E, Rana R, Higgins N (2023). Natural language processing in electronic health records in relation to healthcare decision-making: a systematic review. Comput Biol Med.

[10] Woo K, Adams V, Wilson P (2021). Identifying urinary tract infection–related information in home care nursing notes. J Am Med Dir Assoc.

[11] Topaz M, Koleck TA, Onorato N, Smaldone A, Bakken S (2021). Nursing documentation of symptoms is associated with higher risk of emergency department visits and hospitalizations in homecare patients. Nurs Outlook.

[12] Wieland-Jorna Y, van Kooten D, Verheij RA, de Man Y, Francke AL, Oosterveld-Vlug MG (2024). Natural language processing systems for extracting information from electronic health records about activities of daily living. A systematic review. JAMIA Open.

[13] Trinh VQN, Zhang S, Kovoor J (2023). The use of natural language processing in detecting and predicting falls within the healthcare setting: a systematic review. Int J Qual Health Care.

[14] Mitha S, Schwartz J, Hobensack M (2023). Natural language processing of nursing notes: an integrative review. Comput Inform Nurs.

[15] Zhang Z, Momeni Nezhad MJ, Bagher Hosseini SM (2026). Advancing healthcare with large language models: a scoping review of applications and future directions. Int J Med Inform.

[16] Lai X, Lai Y, Chen J, Huang S, Gao Q, Huang C (2025). Evaluation strategies for large language model-based models in exercise and health coaching: scoping review. J Med Internet Res.

[17] Peters MDJ, Godfrey CM, Khalil H, McInerney P, Parker D, Soares CB (2015). Guidance for conducting systematic scoping reviews. Int J Evid Based Healthc.

[18] Tricco AC, Lillie E, Zarin W (2018). PRISMA extension for scoping reviews (PRISMA-ScR): checklist and explanation. Ann Intern Med.

[19] Evans RS, Benuzillo J, Horne BD (2016). Automated identification and predictive tools to help identify high-risk heart failure patients: pilot evaluation. J Am Med Inform Assoc.

[20] Tang V, Siu PKY, Choy KL (2019). An adaptive clinical decision support system for serving the elderly with chronic diseases in healthcare industry. Expert Systems.

[21] Boitano LT, DeVivo G, Robichaud DI (2023). Successful implementation of a nurse-navigator-run program using natural language processing identifying patients with an abdominal aortic aneurysm. J Vasc Surg.

[22] Hunter J, Freer Y, Gatt A, Reiter E, Sripada S, Sykes C (2012). Automatic generation of natural language nursing shift summaries in neonatal intensive care: BT-Nurse. Artif Intell Med.

[23] Waltzman M, Ozonoff A, Fournier KA (2024). Surveillance of health care-associated violence using natural language processing. Pediatrics.

[24] Nguyen K, Springob P, Evans A (2025). A safety catch system for patient self-service appointment booking. NEJM Catal.

[25] Gilart E, Bocchino A, Gilart-Cantizano P (2025). The integration of AI into the nursing process: a comparative analysis of NANDA, NOC, and NIC-based care plans. Nurs Rep.

[26] Greenhalgh T, Wherton J, Papoutsi C (2017). Beyond adoption: a new framework for theorizing and evaluating nonadoption, abandonment, and challenges to the scale-up, spread, and sustainability of health and care technologies. J Med Internet Res.

[27] Tsai CH, Eghdam A, Davoody N, Wright G, Flowerday S, Koch S (2020). Effects of electronic health record implementation and barriers to adoption and use: a scoping review and qualitative analysis of the content. Life (Basel).

[28] von Gerich H, Moen H, Block LJ (2022). Artificial intelligence -based technologies in nursing: a scoping literature review of the evidence. Int J Nurs Stud.

[29] Abdelmohsen SA, Al-Jabri MM (2025). Artificial intelligence applications in healthcare: a systematic review of their impact on nursing practice and patient outcomes. J Nurs Scholarsh.

[30] Banning M (2008). Clinical reasoning and its application to nursing: concepts and research studies. Nurse Educ Pract.

[31] Shortliffe EH, Sepúlveda MJ (2018). Clinical decision support in the era of artificial intelligence. JAMA.

[32] Topol EJ (2019). High-performance medicine: the convergence of human and artificial intelligence. Nat Med.

[33] Skitka LJ, Mosier KL, Burdick M (1999). Does automation bias decision-making?. Int J Hum Comput Stud.

[34] Albouq SS, Sen AAA, Almashf N, Yamin M, Alshanqiti A, Bahbouh NM (2022). A survey of interoperability challenges and solutions for dealing with them in IoT environment. IEEE Access.

[35] Sharma A, Bhushan K (2026). A comprehensive survey on IoT security: challenges, security issues, and countermeasures. Comput Sci Rev.

[36] London AJ (2019). Artificial intelligence and black-box medical decisions: accuracy versus explainability. Hastings Cent Rep.

