# Supplementary material for: Natural Language Processing in Nursing Practice for Clinical Reasoning and Workflow: Scoping Review
Source: JMIR Nurs. 2026 Oct 16;9:e94778. doi: 10.2196/94778 (PMC13674688; doi:10.2196/94778)
Supplement: Supplementary file 1 [file nursing-v9-e94778-s001.docx]

Multimedia Appendix 1. Detailed search strings and strategies used for PubMed, CINAHL, and other academic databases.

| **Database** | **Concept** | **Search terms / Strategies** |
| --- | --- | --- |
| PubMed | NLP / Text mining | #1 ("Natural Language Processing"[MeSH] OR "Natural Language Processing"[tiab])  #2 ("text mining"[MeSH] OR "text mining"[tiab] OR "data mining"[MeSH] OR "data mining"[tiab])  #3 #1 OR #2 |
|  | Nursing | #4 ("Nursing Care"[MeSH] OR "Nursing Services"[MeSH] OR "Nursing Records"[MeSH] OR "Nursing Assessment"[MeSH] OR nurse*[tiab] OR nursing*[tiab]) |
|  | Final combination | #5 #3 AND #4 |
| CINAHL | NLP / Text mining | S1 (MH "Natural Language Processing")  S2 TI "Natural Language Processing" OR AB "Natural Language Processing"  S3 S1 OR S2  S4 (MH "Text mining")  S5 TI "Text mining" OR AB "Text mining"  S6 TI "Data mining" OR AB "Data mining"  S7 (MH "Data mining")  S8 S4 OR S5 OR S6 OR S7  S9 S3 OR S8 |
|  | Nursing | S10 (MH "Nursing Care+") OR (MH "Nursing Service") OR (MH "Nursing Records") OR (MH "Nursing Assessment")  S11 TI nurse* OR AB nurse* OR TI nursing* OR AB nursing*  S12 S10 OR S11 |
|  | Final combination | S13 S9 AND S12 |
| Cochrane Library | NLP / Text mining | #1 MeSH descriptor: [Natural Language Processing] explode all trees  #2 ("Natural Language Processing"):ti,ab  #3 #1 OR #2  #4 MeSH descriptor: [Data Mining] explode all trees  #5 ("data mining"):ti,ab  #6 #4 OR #5  #7 #3 OR #6 |
|  | Nursing | #8 MeSH descriptor: [Nursing Care] explode all trees  #9 MeSH descriptor: [Nursing Services] explode all trees  #10 MeSH descriptor: [Nursing Records] explode all trees  #11 MeSH descriptor: [Nursing Assessment] explode all trees  #12 (nurse*):ti,ab OR (nursing*):ti,ab  #13 #8 OR #9 OR #10 OR #11 OR #12 |
|  | Final combination | #14 #7 AND #13 |
| Scopus (INDEXTERMS / TITLE-ABS) | NLP / Text mining | #1 INDEXTERMS("Natural Language Processing") OR TITLE-ABS("Natural Language Processing")  #2 INDEXTERMS("text mining") OR TITLE-ABS("text mining")  #3 INDEXTERMS("Data mining") OR TITLE-ABS("Data mining")  #4 #1 OR #2 OR #3 |
|  | Nursing | #5 INDEXTERMS ( "Nursing Care" ) OR INDEXTERMS ( "Nursing Services" ) OR INDEXTERMS ( "Nursing Records" ) OR INDEXTERMS ( "Nursing Assessment" ) OR TITLE-ABS ( nurse* ) OR TITLE-ABS ( nursing* ) |
|  | Final combination | #6 #4 AND #5 |
| Embase | NLP / Text mining | #1 'natural language processing'/exp OR 'natural language processing':ti,ab  #2 'text mining'/exp OR 'text mining':ti,ab  #3 'data mining'/exp OR 'data mining':ti,ab  #4 #1 OR #2 OR #3 |
|  | Nursing | #5 'nursing'/exp OR nurse*:ti,ab OR nursing*:ti,ab |
|  | Final combination | #6 #4 AND #5 |
| Ichushi-Web | NLP / Text mining | #1 自然言語処理/TH OR 自然言語/TA OR "Natural Language Processing"/TA  #2 データマイニング/TH OR "Text mining"/TA  #3 #1 OR #2 |
|  | Nursing | #4 看護ケア/TH OR 看護業務/TH OR 看護記録/TH OR 看護アセスメント/TH OR 看護/TA |
|  | Final combination | #5 #3 AND #4 |
