# Supplementary material for: Natural Language Processing in Nursing Practice for Clinical Reasoning and Workflow: Scoping Review
Source: JMIR Nurs. 2026 Oct 16;9:e94778. doi: 10.2196/94778 (PMC13674688; doi:10.2196/94778)
Supplement: Supplementary file 2 [file nursing-v9-e94778-s002.docx]

Multimedia Appendix 2. Standardized form for data extraction, including clinical settings, NLP techniques, and human-in-the-loop mechanisms.

| **Author (Year)** | **Country / Setting** | **Study design** | **Data source** | **NLP method** | **Nursing practice domain** | **Stage of nursing process** | **Validation & performance** | **Role of NLP** | **Human intervention** | **Reported outcomes / impacts** | **Reported limitations** |
| --- | --- | --- | --- | --- | --- | --- | --- | --- | --- | --- | --- |
|  |  |  |  |  |  |  |  |  |  |  |  |
