# Supplementary material for: Natural Language Processing in Nursing Practice for Clinical Reasoning and Workflow: Scoping Review
Source: JMIR Nurs. 2026 Oct 16;9:e94778. doi: 10.2196/94778 (PMC13674688; doi:10.2196/94778)
Supplement: Supplementary file 3 [file nursing-v9-e94778-s003.docx]

**Multimedia Appendix 3. Characteristics of Included Studies**

| **Author (Year)** | **Country / Setting** | **Target Patients / Clinical Domains** | **Study Design** | **Data Sources** |
| --- | --- | --- | --- | --- |
| Hunter et al. (2012) [22] | UK / Neonatal Intensive Care Unit (Inpatient) | Neonates | Prospective implementation and evaluation study. | Comprehensive electronic patient records (physiological data, intervention logs, and partial free-text nursing notes). |
| Evans et al. (2016) [19] | USA / Tertiary Emergency Hospital (Inpatient) | HF | Pre–post implementation study. | Comprehensive electronic health records (physiological data, intervention records, and dictated reports). |
| Tang et al. (2019) [20] | Taiwan / Long-term Care Facility | Chronic diseases in older adults | Pilot implementation study (system development and field evaluation). | Historical nursing records, admission documents, and web-mined up-to-date medical information. |
| Boitano et al. (2022) [21] | USA / Academic Health System (Population Surveillance) | AAA | Single-center retrospective and prospective implementation study. | Radiology reports within the EHR (CT, MRI, and ultrasound). |
| Waltzman et al. (2024) [23] | USA / Pediatric Tertiary and Community Hospitals (Inpatient) | HAV | Retrospective cross-sectional study with prospective surveillance and external validation. | Nursing notes (handover records and incident-related documentation). |
| Nguyen et al. (2025) [24] | USA / Integrated Healthcare System (Outpatient) | Safety screening | Observational implementation study. | Free-text EHR notes (appointment scheduling messages). |

Abbreviations: AAA, abdominal aortic aneurysm; EHR, electronic health record; HAV, healthcare-associated violence; HF, heart failure; UK, United Kingdom; USA, United States of America.
