# Supplementary material for: Natural Language Processing in Nursing Practice for Clinical Reasoning and Workflow: Scoping Review
Source: JMIR Nurs. 2026 Oct 16;9:e94778. doi: 10.2196/94778 (PMC13674688; doi:10.2196/94778)
Supplement: Supplementary file 4 [file nursing-v9-e94778-s004.docx]

**Multimedia Appendix 4. Application of Natural Language Processing (NLP) in Nursing Practice**

| **Group (Type)** | **Author**  **(Year)** | **Data Acquisition (Trigger)** | **Role of NLP (AI Task)** | **Human-in-the-Loop Mechanisms to Safeguard Clinical Judgment** | **Direct Impact on Nursing Practice** | **Reported Challenges and Limitations** |
| --- | --- | --- | --- | --- | --- | --- |
| Type 1: Text Summarization and Generation | Hunter et al. (2012) [22] | 24-hour nursing summaries (cumulative, summary-oriented) | Automated summarization of structured data and free-text notes | Clinician-led review (primarily by nurses) of generated summaries to assess accuracy, readability, and clinical appropriateness before use in nursing handovers | Nursing handovers and clinical information sharing | The system was limited to specific domains (e.g., respiratory and cardiovascular parameters), emphasizing challenges of developing a system that comprehensively captures all patient status aspects. |
|  | Tang et al. (2019) [20] | Admission assessments (initial, care plan-driven) | Initial care plan generation and semantic matching with the latest clinical evidence | Approval and modification of generated care plans by nurses before implementation | Nursing care planning and chronic disease management | Ensuring patient privacy, protection, and data security when integrating external information sources remains a key challenge. |
| Type 2: Information Extraction and Classification | Evans et al. (2016) [19] | Comprehensive EHR records (longitudinal, integrated) | Automated identification of high-risk patients based on keywords and context | Review and confirmation of NLP-identified high-risk patients by heart failure specialist nurses before care planning | Identification of high-risk patients and clinical decision support | Conducted in a single institution; further validation across diverse clinical settings is required. |
|  | Boitano et al. (2022) [21] | Radiology reports (event-specific, diagnosis-dependent) | Automated identification and surveillance of patients with AAA from radiology reports | Clinical vetting and oversight by nurse navigators | Population health management and nurse navigation | Patients cannot be identified if AAAs are not explicitly documented; the system also relies heavily on dedicated nursing staff. |
|  | Waltzman et al. (2024) [23] | Nursing progress notes (unstructured, narrative) | Machine learning-based extraction and classification of violent incidents | Manual review and performance validation by an expert team | Safety management and prevention of HAV | Manual staff review is required to exclude false positives; fine-grained distinctions in safety-related contexts remain difficult. |
| Type 3: Real-Time Detection and Screening | Nguyen et al. (2025) [24] | Appointment notes and triage records (real-time, triage-oriented) | Real-time detection and flagging of patient safety concerns | Telephone follow-up and clinical assessment by nurses | Telephone nursing triage and patient safety management | Limited infrastructure for longitudinal indicator tracking; false positives owing to insufficient contextual understanding may increase cognitive burden, and post-triage demand may be concentrated in emergency departments. |

Abbreviations: AAA, abdominal aortic aneurysm; EHR, electronic health record; HAV, healthcare-associated violence; HF, heart failure.
